# Downregulation of exosomal let-7a-5p in dust exposed- workers contributes to lung cancer development

**DOI:** 10.1186/s12931-018-0949-y

**Published:** 2018-11-29

**Authors:** Lin Zhang, Changfu Hao, Ruonan Zhai, Di Wang, Jianhui Zhang, Lei Bao, Yiping Li, Wu Yao

**Affiliations:** 1 Department of Occupational Hygiene, School of Public Health and Management, Healthy Shandong Collaborative Innovation Center for Major Social Risk Prediction and Governance, Weifang Medical University, 7166 Baotong West Street, Weifang, 261024 China; 2 Department of Occupational and Environmental Health, School of Public Health, Zhengzhou University, 100 Science Avenue, Zhengzhou, 450001 China

**Keywords:** Pneumoconiosis, Lung cancer, Dust exposure, Exosome, Let-7a-5p

## Abstract

**Background:**

Either chronic or acute exposure to dust particles may lead to pneumoconiosis and lung cancer, and lung cancer mortality among patients diagnosed with pneumoconiosis is increasing. Utilizing genome-wide sequencing technology, this study aimed to identify methods to decrease the number of patients with pneumoconiosis who die from lung cancer.

**Methods:**

One hundred fifty-four subjects were recruited, including 54 pneumoconiosis patients and 100 healthy controls. Exosomes were isolated from the venous blood of every subject. Distinctive miRNAs were identified using high throughput sequencing technology, and bioinformatics analysis predicted target genes involved in lung cancer as well as their corresponding biological functions. Moreover, cross-cancer alterations of genes related to lung cancer were investigated, and survival analysis was performed using 2437 samples with an average follow-up period of 49 months.

**Results:**

Let-7a-5p was revealed to be downregulated by 21.67% in pneumoconiosis. Out of the 683 let-7a-5p target genes identified from bioinformatics analysis, four genes related to five signaling pathways were confirmed to be involved in lung cancer development. Alterations in these four target genes were then explored in 4105 lung cancer patients, and BCL2L1 and IGF1R were demonstrated to be aberrantly expressed. Survival analysis further revealed that high expression of BCL2L1 corresponded to reduced survival of lung cancer patients (HR (95%CI) = 1.75(1.33~2.30)), while patient survival time was unaffected by expression of IGF1R (HR (95%CI) = 1.15 (0.98~1.36)).

**Conclusions:**

In patients with lung adenocarcinoma, simultaneous downregulation of exosomal let-7a-5p and elevated expression of BCL2L1 are useful as predictive biomarkers for poor survival.

## Background

Exposure to dust particles is implicated in the etiology of both pneumoconiosis and lung cancer [1, 2], and the relationship between pneumoconiosis and lung cancer has drawn considerable attention. Researchers have proposed that patients suffering from pneumoconiosis are likely at high risk for developing lung cancer. As reported in previous studies, the mortality O/E ratio of silicosis is as high as 6.03 compared to the general population [3], and a pooled exposure-response analysis of 10 silica-exposed cohorts conducted by the International Agency for Research on Cancer (IARC) further confirmed increased risk of lung cancer in patients diagnosed with pneumoconiosis [4]. Meanwhile, increasing cases of pneumoconiosis patients occurs every year in China, India, and other developing countries. Official data released by the National Health and Family Planning Commission of China showed where 26,081 cases of pneumoconiosis were reported in 2015, comprising 93.92% of all newly diagnosed occupational diseases that year. The patient number has increased in the last five consecutive years. Therefore, it is of great significance to control/decrease the number of patients with pneumoconiosis who die from lung cancer.

Development of pneumoconiosis is a complex biological process in which intercellular communication plays essential roles [5]. Exosomes have emerged as efficient vectors for intercellular cargo transportation [6]. Being-transported as exosome cargo, microRNAs (miRNAs) have been demonstrated/validated as crucial regulators in many diseases [7]. For example, exosomal let-7a-5p and let-7i-5p were found to be associated with nanoparticle phagocytosis. Both of these factors simultaneously target WASL and VASP inducing inflammatory responses in lung tissue [8]. Moreover, let-7a was shown to be differentially expressed in different subtypes of non-small cell lung cancer [9–11]. Therefore, let-7 families may be useful as specific biomarkers for distinguishing different subtypes of lung cancer.

Considering the critical role of exosomal let-7a-5p in both pneumoconiosis and lung cancer, the purpose of this study was to predict the risk of lung cancer in patients with pneumoconiosis using exosomal let-7a-5p. Expression of let-7a-5p in exosomes isolated from pneumoconiosis patient serum was first investigated. Subsequently, target genes of let-7a-5p were predicted and validated, in which genes related to lung cancer were screened for further analysis. After comparing gene expression between lung cancer and control samples, survival analysis was performed on a large patient sample diagnosed with lung cancer. The results of this study may contribute to identify biomarkers for lung cancer in pneumoconiosis patients.

## Methods

### Subjects

One hundred fifty-four subjects, including 54 pneumoconiosis patients and 100 healthy controls, were recruited from Henan Provincial Institute of Occupational Health, China. Pneumoconiosis severity was graded according to the National Diagnostic Criteria for Pneumoconiosis of China (GBZ 70–2002). Male workers undergoing preservice physical examinations served as healthy controls. Subjects with disorders that may affect exosome excretion were excluded. Five milliliters of venous blood were obtained from each subject, and exosomes were isolated from serum. All subjects involved in this study were well informed about the purpose of this study, and the research protocol was approved by the institutional review board of Zhengzhou University.

### Isolation and characterization of exosomes

Standard methods for exosome isolation and characterization have been previously described [8]. Briefly, five milliliters mixed serum was first diluted with 20-fold volume PBS and centrifuged at 2000 × g, 4 °C for 30 min. Next, the supernatant was collected and centrifuged at 12000 × g, 4 °C for 45 min to exclude pellets. The supernatant was then centrifuged against 110,000 g for 2 h (70 Ti centrifuge rotor, Beckman Coulter Inc., USA), and the raw exosomes at the bottom of the ultracentrifuge tube were collected and diluted with 20 mL PBS. After filtering with a 0.22 μm filter, the suspension was centrifuged for 70 min at 110,000 × g, the pellet was collected, and this step was repeated an additional time. Finally, the pellet of exosomes at the bottom of the centrifuge tube was resuspended in 100 μl PBS. For exosome morphological characterization and quantification, we used transmission electron microscopy (TEM) (HT7700, Hitachi, Japan) and nanoparticle tracking analysis (NTA) (NanoSight® LM10, Malvern, UK).

### Real-time quantitative PCR (RT-qPCR) for exosomal miRNA detection

Total exosomal miRNA was first isolated from the serum of all subjects, and cDNA was obtained using a miRNA reverse kit (Sangon Biotech, China). Small nuclear RNA U6 was selected as an internal control for RT-qPCR. RT-qPCR was conducted using SYBR Green (Sangon Biotech, China) with a StepOnePlus Real-Time PCR System (Applied Biosystems, USA). Primers for RT-qPCR are as follows: let-7a-5p, 5′-TGAGGTAGTAGGTTGTATAGTT-3′; U6-S, 5′-CTCGCTTCGGCAGCACA-3′; U6-A, 5′-AACGCTTCACGAATTTGCGT-3′.

### Target gene prediction and functional annotation for exosomal let-7a-5p

Target genes of let-7a-5p were predicted using the MR-microT method (available online at http://diana.imis.athena-innovation.gr/DianaTools) [12], which provides near real-time predictions for custom miRNA sequences, and genes with a miTG score ≥ 0.8 were selected for further investigation. For miRNA functional annotation, Gene Ontology (GO) analysis and Kyoto Encyclopedia of Genes and Genomes (KEGG) pathway enrichment were conducted using DAVID 6.8 online (https://david.ncifcrf.gov/) [13], ClueGo v2.3.5, and CluePedia v1.3.5 (thresholds account ≥2 and EASE score ≤ 0.1).

### Identification of let-7a-5p target genes related to lung cancer

All target genes and signaling pathways identified in the last step were aligned with items confirmed in lung cancer using the MalaCards platform, an integrated database of human maladies and their annotations modeled on the architecture and richness of the popular GeneCards database of human genes available online at http://www.malacards.org/ [14]. The interactive items enriched from the alignments were identified using the intersection of a Venn diagram and selected for downstream analysis.

### Cross-cancer alteration of let-7a-5p target genes

To investigate alterations of four target genes of let-7a-5p in lung cancer, we explored their expression in lung cancer (lung adenocarcinoma (LA) and lung squamous carcinoma (LSC)) using TCGA database. Four types of genomic alterations, including mutation, deletion, amplification, and multiple alterations, were identified, respectively. Moreover, expression levels of these genes in both LA and LSC were used to generate a heat map.

### Kaplan-Meier and cox hazards regression survival analysis

To investigate the relationship between expression of BCL2L1 and IGF1R and survival of patients diagnosed with lung cancer, we conducted Kaplan-Meier and Cox hazards regression survival analysis using the online database registered as KM plotter for lung cancer [15]. The database integrates 54,675 genes on survival using 10,461 cancer samples, including 2437 lung cancer patients with a mean follow-up time of 49 months. Additionally, to examine the effect of aberrantly expressed BCL2L1 and IGF1R on different subtypes of lung cancer, survival analysis was conducted on patients diagnosed with LA and LSC, respectively.

### Comparison of differential BCL2L1 and IGF1R expression between lung cancer and adjacent normal control tissues

Expression of BCL2L1 and IGF1R in lung cancer and adjacent normal control tissues were investigated and compared using the Oncomine database premium version, an online platform that integrates 715 datasets and 86,733 samples and has been widely used for cancer research [16]. Results were shown using boxplots.

### Statistical analysis

Data were analyzed using SAS 9.2 for Windows (SAS Institute Inc., Cary, NC, USA) and are expressed as the mean ± standard deviation (SD). Student’s *t*-test was used for comparisons between two independent samples. A *P* value less than 0.05 was considered to be statistically significant unless otherwise indicated.

## Results

### Characterization of exosomes and quantification of exosomal let-7a-5p

**Fig. 1:**
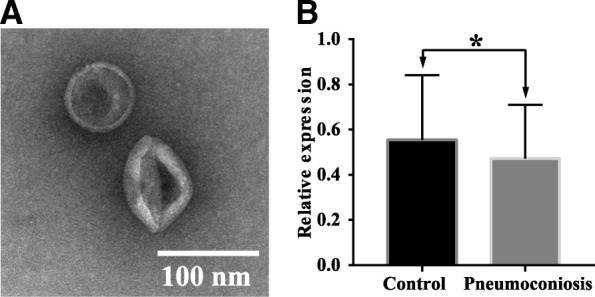
Characterization of exosomes and quantification of exosomal let-7a-5p. **a** Morphological characterization of exosomes using transmission electron microscopy. Exosomes appeared as saucer-like vesicles with diameters between 30 and 150 nm and clear bilayer plasmalemma. **b** Expression of exosomal let-7a-5p in pneumoconiosis patients and healthy controls, showing that let-7a-5p is downregulated in pneumoconiosis patients compared to healthy controls

Under TEM, exosomes were observed as saucer-like vesicles with diameters between 30 and 150 nm with clear bilayer plasmalemma (Fig. 1a). Expression of exosomal let-7a-5p was quantified by Ploy (A) tailing and RT-qPCR amplification. Compared to healthy controls, relative expression levels of exosomal let-7a-5p in venous blood from pneumoconiosis patients was decreased by 21.67% (0.47 ± 0.24 vs. 0.60 ± 0.24, *t* = 3.11, *P* = 0.002) (Fig. 1b), consistent with high throughput sequencing results shown in our previous study [8], suggesting that exosomal let-7a-5p may be involved in the development of pneumoconiosis.

### Target gene prediction and functional annotation for exosomal let-7a-5p

**Fig. 2:**
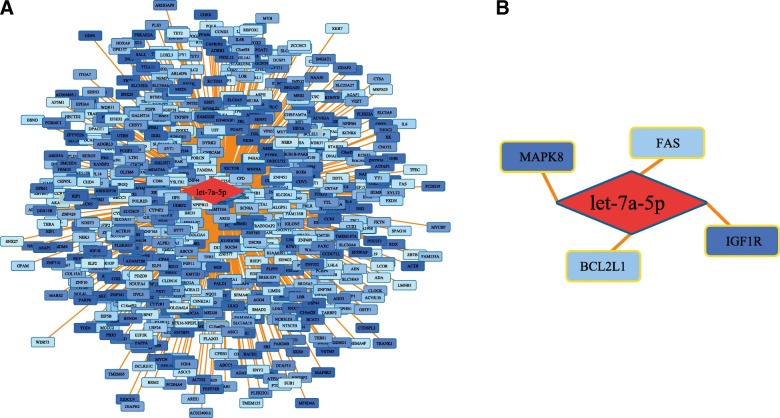
Prediction of target genes for exosomal let-7a-5p. **a** Six hundred eighty three target genes of exosomal let-7a-5p were predicted using the MR-microT method, and genes that were validated are represented using dark blue color, while nonvalidated genes were shown using light blue color. **b** To identify target genes of exosomal let-7a-5p, all potential target genes were aligned with the MalaCards platform, in which four target genes were determined, including BCL2L1, FAS, MAPK8, and IGF1R

**Fig. 3:**
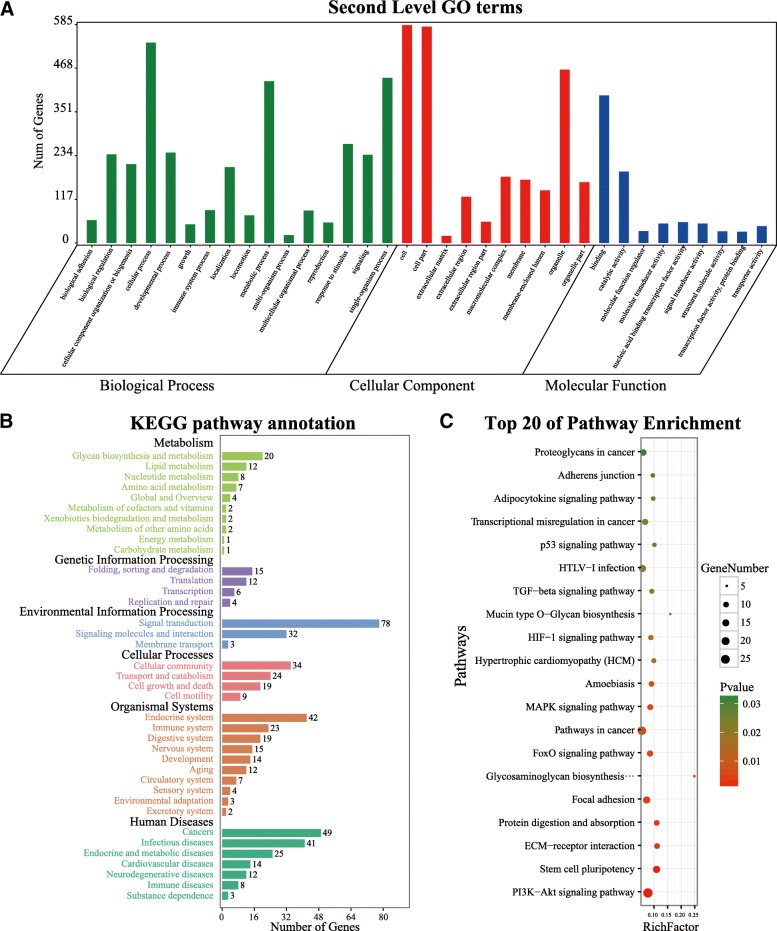
Functional annotation for let-7a-5p. The biological function of exosomal let-7a-5p was investigated using gene ontology (GO) analysis and Kyoto Encyclopedia of Genes and Genomes (KEGG) pathway enrichment. **a** Results of GO analysis revealed from three features: biological process (green), cellular component (red), and molecular function (blue). Signaling pathways regulated by exosomal let-7a-5p from a total of six aspects are shown in **b**, and the top 20 signaling pathways are listed in **c**

Six hundred eighty-three target genes of let-7a-5p were identified and used for downstream functional annotation (Fig. 2), including biological process, molecular function, cellular component, and signaling pathway (Fig. 3). As shown, the predicted target genes of let-7a-5p were related to cellular metabolism, cellular protein modification, cell differentiation, and cell cycle. For cellular component, eight of the predicted genes code for proteins that play important roles in the cytoplasm, endomembrane, and lumen. In addition, 26 signaling pathways were identified, in particular, signaling pathways such as PI3K-Akt, AMPK, and TGF-beta, which have been widely validated in pneumoconiosis, lung cancer, and other respiratory diseases.

### Screening for target genes and signaling pathways related to lung cancer

All target genes and signaling pathways obtained above were aligned with items related to lung cancer using the MalaCards platform, and interactive items were selected for further analysis. Four target genes (BCL2L1, IGF1R, MAPK8, and FAS) and 5 signaling pathways (PI3K-Akt, FoxO, MAPK, pathways in cancer, and proteoglycans in cancer) were identified. Interestingly, all four target genes were covered, or at least partially covered, by the five signaling pathways identified: BCL2L1 and IGF1R are involved in PI3K-Akt signaling, IGF1R and MAPK8 in FoxO signaling, IGF1R and FAS in proteoglycans in cancer, and MAPK8 and FAS in MAPK signaling. All of these genes are involved in cancer pathways, suggesting critical roles for these four target genes in the development of lung cancer.

### Alterations of four target genes of exosomal let-7a-5p in lung cancer

**Fig. 4:**
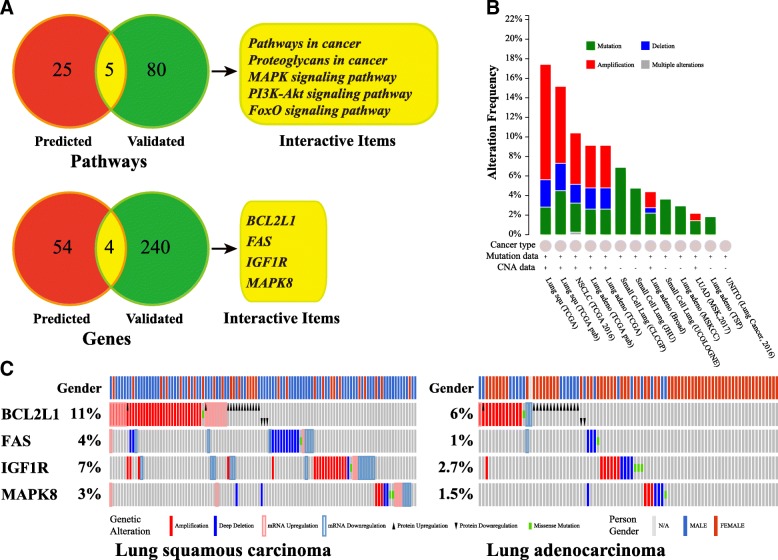
Identification of target genes and signaling pathways related to lung cancer for exosomal let-7a-5p. **a** Two Venn diagrams showing signaling pathways and target genes of exosomal let-7a-5p related to lung cancer. The intersection of prediction and validation for both signaling pathways and target genes were determined and used as the main regulatory pattern of exosomal let-7a-5p. **b** and **c** represent alterations of lung cancer-related target genes that were investigated in 4105 lung cancer patients

Expression and alterations of BCL2L1, IGF1R, MAPK8, and FAS were investigated in lung cancer using TCGA database, and 4105 clinical samples provided by 13 studies were utilized in this study (Fig. 4). Mutations in these four target genes were widely distributed among all clinical subtypes of lung cancer, while amplifications were mainly found in LSC, LA, and other types of non-small cell lung cancer. BCL2L1 was amplified and upregulated in both LSC (11%) and LA (6%), but there was no statistical difference between males and females. Alterations of IGF1R were present in 7% LSC samples, which were mainly amplified and up-regulated. However, alterations of MAPK8 and FAS were present in less than 5% of samples. Therefore, only BCL2L1 and IGF1R were selected for further investigation.

### Association between expression of BCL2L1 and IGF1R and survival of lung cancer patients

**Fig. 5:**
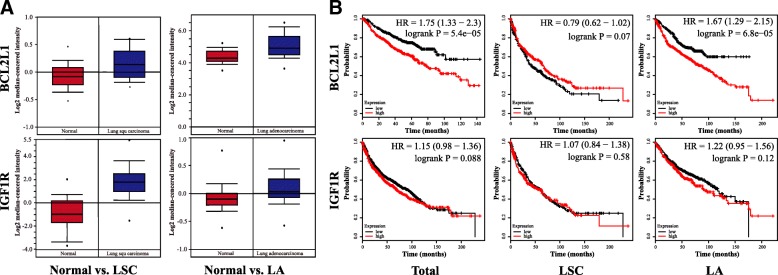
Expression comparison and survival analysis of BCL2L1 and IGF1R. **a** Expression comparison of BCL2L1 and IGF1R between lung cancer and adjacent normal control tissues. These two genes are upregulated in both lung adenocarcinoma (LA) and lung squamous carcinoma (LSC). **b** Survival analysis of patients diagnosed with lung cancer. High BCL2L1 expression levels are correlated with poor survival in lung adenocarcinoma patients. In contrast, no correlation was found between IGF1R expression and patient survival in either LA or LSC

Expression of BCL2L1 and IGF1R was first investigated using the Oncomine database (Fig. 5a). Compared to adjacent normal control tissue, expression of these two genes in both LA and LSC was statistically increased (Table 1). One thousand nine hundred twenty-six patients with lung cancer were then recruited to study the association between differential expression of BCL2L1 and IGF1R and patient survival using Kaplan-Meier and Cox regression method (Fig. 5b). Results suggested that high levels of BCL2L1 decease the survival of lung cancer patients (HR (95%CI) = 1.75(1.33~2.30)), while patients survival time was unaffected by expression of IGF1R (HR (95%CI) = 1.15(0.98~1.36)). For different subtypes of lung cancer, high expression of BCL2L1 correlates with reduced survival of LA patients (HR (95%CI) = 1.67(1.29~2.15)), but it did not affect survival time of patients with LSC (HR (95%CI) =0.79(0.62~1.02)). Thus, inhibition of BCL2L1 by exosomal let-7a-5p may be involved in LA.

**Table 1:** Comparison of differential expression of BCL2L1 and IGF1R in lung cancer vs. adjacent normal control tissues

Variable	BCL2L1	IGF1R
Lung Squamous Carcinoma		
Source	Hou	Bhattacharjee
N	156	203
Fold Change	1.18	7.04
*t*	3.88	5.62
*p*	< 0.001	< 0.001
Lung Adenocarcinoma		
Source	Selamat	Hou
N	116	156
Fold Change	1.62	1.11
*t*	6.29	2.99
*p*	< 0.001	0.002

## Discussion

In this study, total exosomes were first isolated from the serum of pneumoconiosis patients. Expression of exosomal let-7a-5p was measured using RT-qPCR and was found to be downregulated compared to the normal controls. Previous studies have shown that a certain number of miRNAs of let-7 families were also downregulated in the majority of non-small cell lung cancer. For instance, downregulation of let-7a-2-3p attenuated the inhibition of K-ras and promoted radon induced experimental lung cancer [17]. In addition, expression of let-7a is also significantly reduced in the serum of non-small cell lung cancer [9]. As exosomal let-7a-5p expression is similarly expressed in LC and pneumoconiosis patients, targeting let-7a-5p may be a useful strategy to decrease the number of patients with pneumoconiosis who die from lung cancer.

Intensive investigation into the role of exosomal let-7a-5p on lung cancer development will contribute to a decrease in the number of LC deaths caused by exposure to silica dust. Using bioinformatics tools, we identified let-7a-5p target genes possibly involved in lung cancer and pneumoconiosis. Four target genes, BCL2L1, IGF1R, MAPK8, and FAS, regulated by exosomal let-7a-5p were identified. One of these genes, BCL2L1, is known to collaborate with SOX2 to promote cell proliferation in lung cancer [18]; nuclear translocation of IGF1R induced growth arrest and apoptosis resistance of lung cancer cells [19]. MAPK8 is associated with the production and elimination of reactive oxygen species (ROS), while FAS mainly participates in cellular apoptosis in lung cancer [20]. For signaling pathways, PI3K-Akt, FoxO, proteoglycans in cancer, and MAPK have also been reported in lung cancer in previous studies [21–26]. Thus, the identified putative target genes and signaling pathways were highly precise and could be used for further analysis.

To investigate expression of genes targeted by exosomal let-7a-5p that are also related to lung cancer, alterations of BCL2L1, IGF1R, MAPK8, and FAS were explored in 4105 clinical lung cancer samples. Changes, such as amplification and mutation, were found in these genes in both LA and LSC, and expression of all these four target genes was subsequently investigated. As a result, expression of BCL2L1 and IGF1R were found to be increased in all lung cancer subtypes. In accordance with previous studies, activation of IGF1R contributed to the resistance of radiotherapy in lung cancer cells [27]; however, no data on BCL2L1 expression in LA or LSC is yet available to our knowledge.

The effect of differential expression of BCL2L1 and IGF1R on survival time of lung cancer patients was also investigated. First, we compared the expression of these two genes between lung cancer patients and normal controls. As shown in Table 1, expression of IGF1R was upregulated by 7.04-fold in LSC, and BCL2L1 was upregulated by 1.65-fold in LA. Then, we analyzed the relationship between expression of these two genes and survival in lung cancer patient, revealing that survival was correlated with expression of BCL2L1 (HR (95%CI) = 1.75(1.33–2.30)) but not correlated with expression of IGF1R (HR (95%CI) = 1.15(0.98–1.36)). In particular, a strong correlation between the expression of BCL2 L1 and poor patient survival was found in patients with LA (HR (95%CI) = 1.67(1.29, 2.15)) but not in patients with LSC (HR (95%CI) = 0.79(0.62, 1.02)), suggesting that expression of BCL2L1 could be useful as a biomarker for LA patient survival.

## Conclusions

The expression of exosomal let-7a-5p was detected in pneumoconiosis, and all potential target genes related to exosomal let-7a-5p were predicted, among which four target genes related to lung cancer were selected for downstream analysis, including BCL2L1, IGF1R, MAPK8, and FAS. Levels of BCL2L1 were assessed in 4000 clinical samples of diverse subtypes of lung cancer biopsy samples, as well as in controls. Downregulation of let-7a-5p in both pneumoconiosis patients and LA patients may be involved in the onset of LA in patients with pneumoconiosis. Moreover, the strong correlation between downregulation of let-7a-5p and elevated expression of BCL2L1 with LA patient survival suggests that expression levels of those biomarkers can be useful for predicting LA patient survival. Taken together, these results suggest that expression of let-7a-5p in pneumoconiosis is downregulated, while reduced let-7a-5p corresponds to high expression of BCL2L1 and poor survival of LA patients. Thus, this approach may present a novel method to decrease mortality of pneumoconiosis complicated by lung cancer via increasing expression of let-7a-5p.

## Abbreviations

BCL2L1: B-cell lymphoma-2 like 1
CI: confidence interval
GO: gene ontology
HR: hazard ratio
IGF1R: insulin-like growth factor 1 receptor
KEGG: Kyoto Encyclopedia of Genes and Genomes
LA: lung adenocarcinoma
LSC: lung squamous carcinoma
MAPK8: mitogen-activated protein kinase 8
NTA: nanoparticle tracking analysis
ROS: reactive oxygen species
RT-qPCR: Real-time quantitative polymerase chain reaction
SD: standard deviation
TEM: transmission electron microscopy
